# Monoclonal Antibodies: Leading Actors in the Relapsed/Refractory Multiple Myeloma Treatment

**DOI:** 10.3390/ph13120426

**Published:** 2020-11-27

**Authors:** Sonia Morè, Maria Teresa Petrucci, Laura Corvatta, Francesca Fazio, Massimo Offidani, Attilio Olivieri

**Affiliations:** 1 Clinica di Ematologia, Azienda Ospedaliero-Universitaria Ospedali Riuniti di Ancona, 60126 Ancona, Italy; sonia.more@live.it (S.M.); a.olivieri@univpm.it (A.O.); 2 Sezione di Ematologia, Dipartimento di Medicina Traslazionale e di Precisione, Azienda Ospedaliera Policlinico Umberto I, Università “Sapienza” di Roma, 00161 Roma, Italy; petrucci@bce.uniroma.it (M.T.P.); fazio@bce.uniroma1.it (F.F.); 3 UOC Medicina, Ospedale Profili Fabriano, 60044 Fabriano, Italy; laura.corvatta@sanita.marche.it

**Keywords:** relapsed multiple myeloma, elotuzumab, daratumumab, isatuximab

## Abstract

Multiple myeloma is a complex hematologic malignancy, and despite a survival improvement related to the growing number of available therapeutic options since 2000s, it remains an incurable disease with most patients experiencing relapse. However, therapeutic options for this disease are constantly evolving and immunotherapy is becoming the mainstay of the therapeutic armamentarium of Multiple Myeloma (MM), starting with monoclonal antibodies (MoAbs) as elotuzumab, daratumumab and isatuximab. Elotuzumab, the first in class targeting SLAMF7, in combination with lenalidomide and dexamethasone and daratumumab, directed against CD38, in combination with Rd and with bortezomib and dexamethasone (Vd), have been approved for the treatment of relapsed/refractory MM (RRMM) after they demonstrated excellent efficacy. More recently, another anti-CD38 MoAb named isatuximab was approved by FDA in combination with pomalidomide-dexamethasone (Pd) in the same setting. Many phase II and III trials with regimens containing these MoAbs are ongoing, and when available, preliminary data are very encouraging. In this review we will describe the results of major clinical studies that have been conducted with elotuzumab, daratumumab and isatuximab in RRMM, focusing on phase III trials. Moreover, we will summarized the emerging MoAbs-based combinations in the RRMM landscape.

## 1. Introduction

The introduction of high-dose therapy in the 1990s and the development of novel classes of drug since the 2000s, led to a significant improved outcome of MM patients [1,2,3] and recent studies report a 10-year survival until 60% in younger MM patients [4] and a four-year survival of 56% in elderly [5]. However, despite a possible long-lasting remission in some patients, MM remains an incurable disease, and until recently, patients who became non-responsive to immunomodulatory agents (IMiDs) and proteasome inhibitors (PIs) showed an overall survival (OS) of 13 months [6]. These results are consistent with the most recent knowledge on genomic and molecular characterization of MM by next generation sequencing (NGS), showing a lack of universal driver mutation, presence of coexistent subclones and oligoclonality in MM, leading to different type of evolution of the disease over time and drug resistance [7]. Beside biological disease complexity, in patients with relapsed/refractory MM (RRMM) treatment, decisions must take into account two key points. Firstly, the duration of response, progression-free survival (PFS) and OS decrease with successive lines of therapy [8,9], and secondly, the percentage of patients who receive a second-line treatment is 61% as reported by a retrospective European review [10].

Managing RRMM patients can be compared, at present, in order to unravel a skein if we consider all patient-related and disease-related factors to evaluate the choice of therapy in this population. The start of therapy is recommended in patients with clinical relapse as IMWG criteria [11], but it has to also be considered in patients with biochemical relapse, particularly in the presence of a rapid increase in M protein, and specifically when the level of M protein is doubled over two months, having reached at least 1 g/dL in the serum and 0.5 g/24 h in the urine [12,13]. Besides patient factors as age, performance status, frailty, comorbidities, but also patient preference and logistics of drug administration, treatment of relapsed MM should be selected on the basis of disease-related factors. This is because the presence of high-risk features (renal failure, extramedullary MM, high-risk cytogenetic) and therapeutic history, in terms of number of prior lines of therapy, quality and duration of response and toxicity with prior, drugs in order to identify the best approach. Monoclonal antibodies (mAbs) are widely used and represent a breakthrough in the management of hematologic malignancies. Rituximab (mAb against the CD20 antigen) [14], and brentuximab (mAb against CD30 molecules) [15], have significantly improved the outcome of patients with B cell lymphomas and Hodgkin lymphoma. Until now, elotuzumab, mAb targeting SLAM7 in the plasma cells, and daratumumab, mAb binding CD38 molecule, have been approved for the treatment of RRMM. Whereas, several studies evaluated and are assessing another mAb targeting CD38, isatuximab. In this review, we summarized the results of trials conducted so far with these mAbs in RRMM.

## 2. Elotuzumab 

### 2.1. Elotuzumab Plus Immunomodulatory Drugs (IMiDs)

Elotuzumab is a humanized immunoglobulin G1 kappa (IgG1), specific for human SLAMF7 and it does not show cross-reactivity with non-human homologues or other signaling lymphocyte activation molecule (SLAM) family members [16]. Elotuzumab is actually approved by Food and Drugs Administration (FDA) for the treatment of RRMM patients, in association with lenalidomide-dexamethasone (Elo-Rd) or pomalidomide-dexamethasone (Elo-Pd).

After a phase I study [17] showed no response in patients with advanced MM receiving elotuzumab as single agent, it was evaluated in combination with IMids, starting with thalidomide. Low efficacy was reported with the combination elotuzumab, thalidomide and low-dose dexamethasone (ETd) in a multicenter phase II study [18] with the primary endpoint of evaluating grade 3–4 non-hematological toxicity. Fifty-one patients with a median of 3 prior lines of therapy received elotuzumab 10 mg/kg weekly for the first two cycles and every two weeks thereafter, thalidomide at escalating doses from 50 mg to 200 mg daily and dexamethasone 40 mg weekly. Grade ≥3 non hematological adverse events occurred in 63% of patients mostly fatigue (35%) and peripheral oedema (25%) whereas 15% of patients had an infusion reaction (IRRs). At least a partial response (PR) was achieved by 38% of patients and median PFS was 3.9 months. 

Triplet including elotuzumab, lenalidomide and dexamethasone (Elo-Rd) has been assessed in phase I dose escalation study [19], whose primary objective was to identify the maximum tolerated dose (MTD) of elotuzumab, administered at escalating dose from 5 to 20 mg/kg weekly for the first 2 cycles and every two weeks thereafter, combined with lenalidomide (25 mg days 1–21) and dexamethasone (40 mg/weekly). Twenty-eight patients with a median of 3 prior therapies were enrolled. The MTD was not reached up the dose of 20 mg/kg and the most common grade 3–4 adverse events were neutropenia (36%), thrombocytopenia (21%), diarrhoea (11%). Eighty-nine percent of patients developed infusion reaction (consisting on pyrexia, nausea, chills, flushing, rash, chest discomfort) during the first infusion of elotuzumab, mainly of grade 1–2 and resolving within 24 h. As regard response to triplet combination Elo-Rd, 82% of patients achieved at least a PR, 32% at least very good partial response (VGPR) and 4% a complete response (CR) and the response was not affected by the number of previous therapies received. In the phase II expansion cohort of the same study [20], patients who had received one to three previous lines of therapy (except for lenalidomide, allowed in the phase I study) were assigned to receive either elotuzumab 10 mg/kg or 20 mg/kg combined with lenalidomide and dexamethasone at the same schedules of phase I study. Among 73 patients enrolled, 44% and 11% had received 2, and 3 previous lines of treatment, respectively. Overall, response rates, the main endpoint of the study, were as follows: 84% at least a PR, 56% at least VGPR and 14% at least CR. After a median follow-up of 21.2 months, median PFS was 28.6 months for all patients (32.4 months for the 10 mg/kg group and 25.0 months for the 20 mg/kg group). IRRs occurred in only 8 patients (11%) and were grade 3 only in one patient. As well as the phase I study, the main grade 3–4 adverse events were neutropenia (19%), thrombocytopenia (18%) and diarrhoea (10%).

Based on these results, the phase 3 trials ELOQUENT-2 [21], compared Elo-Rd versus Rd in 646 RRMM patients with a median age of 66 years and a median of 2 lines of prior therapies (range 1–4). Overall, 70% of patients had received bortezomib, 48% thalidomide, 6% lenalidomide and 35% were refractory to their last line of therapy. Elo-Rd group received elotuzumab 10 mg/kg on days 1, 8, 15, and 22 during the first two cycles and every two weeks thereafter, lenalidomide 25 mg days 1–21 and oral dexamethasone 40 weekly whereas the control group received Rd. After a 4-year follow-up [22], ERd significantly improved PFS versus Rd (median 19.4 months versus 14.9 months; HR = 0.71; *p* = 0.0004). The greatest PFS benefit among the subgroups was observed in patients at the median time or further from diagnosis (≥3.5 years) with 1 prior line of therapy, who had a 44% reduction in the risk of progression/death, and in patients with a high-risk MM, who had a 36% reduction in favor of Elo-Rd. The overall response rate (ORR) was 79% with Elo-Rd versus 66% with Rd and at least VGPR was obtained by 35% of Elo-Rd patients versus 29% of Rd group. Elotuzumab did not add hematological or nonhematological toxicity to Rd besides IRRs occurring in 10% of patients, mainly grade 1–2. After a median follow-up of 70.6 months [23], final analysis the study showed a significant OS benefit in patients receiving Elo-Rd versus Rd since median OS was 48.3 versus 39.6 months in the Rd arm (hazard ratio, HR = 0.82; *p* = 0.04) so ELOQUENT-2 represents the first trial to demonstrate a significant OS advantage with an antibody-based triplet regimen in RRMM. Remarkably, OS benefit was maintained across relevant subgroups of patients as well as ≥75 years old (median 48.5 months versus 27.4 months; HR = 0.69), those with 2–3 prior lines of therapy (median 51 months versus 33.6 months; HR = 0.71) and patients with high-risk cytogenetics (median 29.8 months versus 24.8 months; HR = 0.69) [23]. Recently, Gentile et al. [24] reported data of an Italian real-life experience on Elo-Rd administered to 300 RRMM, 41% of whom aged ≥75 years. The results of this retrospective analysis were consistent with ELOQUENT-2 trial since ORR was 77% and median PFS 17.6 months. 

Elotuzumab was tested in combination with pomalidomide in the randomized phase II ELOQUENT-3 trial [25], demonstrating that the addiction of elotuzumab to the backbone pomalidomide-dexamethasone (Pd) induces a 46% reduction in progression or death. Sixty patients received Elo-Pd (elotuzumab 10 mg/kg on days 1, 8, 15, 22 for 2 cycles, and 20 mg/kg on day 1 for the next 28-day cycles; pomalidomide 4 mg per day on days 1 to 21 of 28-day cycles; dexamethasone 40 mg weekly) and 57 patients received Pd alone. Patients had a median of 3 (range 2–8) previous therapies and, in Elo-Pd group 68% of patients (versus 72% in PD group) were refractory to both bortezomib and lenalidomide. After a median follow-up of 9.1 months, median PFS was 10.3 versus 4.7 months in Elo-Pd versus Pd groups, respectively (HR 0.54, *p* = 0.008). This advantage was preserved in all the subgroups, also in patients with HR cytogenetic and in lenalidomide-refractory ones. The ORR was 53% in elotuzumab group and 26% in Pd group. As regard safety profile, triplet combination demonstrated to provide a substantial clinical benefit without added clinically relevant toxicities. Main adverse events are pictured in Table 1. In 2018 the combination Elo-Pd had the FDA approval for RRMM who had received at least two previous lines of therapy. 

### 2.2. Elotuzumab Plus Proteasome Inhibitors (PIs)

The combination of elotuzumab with a PI was tested in a multicenter randomized phase II study [26] comparing elotuzumab, bortezomib and dexamethasone (Elo-VD), with bortezomib-dexamethasone (VD) in 152 RRMM patients, treated with no more than 3 prior lines of therapy who had not to be bortezomib refractory. Overall, 66% and 34% of patients had received one and 2/3 prior lines of therapy, respectively. In Elo-VD group treatment consisted in elotuzumab (10 mg/kg/weekly for cycles 1 and 2, days 1 and 11 for cycles 3–8 and days 1–15 thereafter), bortezomib (1.3 mg/m^2^ on days 1, 4, 8, 11) and dexamethasone (20 mg on non-elotuzumab dosing days). Whereas, the control arm received VD. The study met the primary endpoint, since PFS was significantly longer with Elo-VD than VD (median 9.7 months versus 6.9 months; HR = 0.72; *p* = 0.09, exceeding the prespecified significance level of 2-sided *p* ≤ 0.3).

Moreover, patients Elo-VD-treated homozygous for the high-affinity FcγRIIIa V allele had a better trend towards longer PFS compared with those VD-treated with the same characteristic (median 22.3 versus 8.2 months) being FcγRIIIa receptor expressed in NK cells and required to bind Fc part of elotuzumab to induce ADCC. No differences were reported between Elo-Vd and VD in terms of ORR (66% versus 63%) and 2-years OS (73% versus 66%; HR 0.75). The rate of patients went off-treatment because of toxicity was similar in the two arms (13%, versus 19%, respectively). More frequent grade 3–4 adverse events was pneumonia, thrombocytopenia, diarrhoea and anemia which were quite similar in the two arms (Table 1). Elotuzumab infusion reaction occurred in 5% of patients, mainly of grade 1–2.

Nordic Myeloma Study Group [32] assessed elotuzumab in combination with carfilzomib, instead of bortezomib, and dexamethasone (EKd) after 1–3 prior treatment lines and preliminary data showed and ORR of 91% using weekly carfilzomib 70 mg/m^2^. Notably, the best responding patients displayed mutation to RAS genes.

Quadruplet elotuzumab-bortezomib-pomalidomide-dexamethasone (Elo-PVd) was studied in a phase 2 trial (NCT02718833) [33] including 48 patients with a median of 3 prior regimens (range 1–9). All patients had received prior lenalidomide, 96% bortezomib, 29% carfilzomib, 33% pomalidomide, 25% daratumumab and were refractory to their last line of therapy. This quadruplet induced an ORR of 61% and a median PFS of 9.8 months. In patients with one prior line of therapy, ORR was 74% and median PFS was not reached. Most frequent grade ≥3 haematological adverse event was neutropenia (29%) whereas grade 3–4 pneumonia occurred in 27% of patiens and were the most common non-hematologic toxicities. Patients who received prior pomalidomide, carfilzomib, and/or anti-CD38 monoclonal antibody also benefited. 

### 2.3. Ongoing Clinical Trials with Elotuzumab in RRMM Patients

An ongoing clinical trial (NCT03030261) is evaluating Elo-Pd as induction and consolidation/maintenace after second ASCT in patients with RRMM. Elo-Pd combination has been compared with elotuzumab in association with PD-1 inhibitor nivolumab (EN) in a phase 2 multiple cohort study (NCT02612779), where enrolling patients relapsed o refractory to prior therapy with lenalidomide. The results are not available yet. 

Other quadruplets including elotuzumab are under investigation in the setting of RRMM. A phase II study (NCT03361306) is assessing the efficacy, in term of VGPR, of the combination elotuzumab, carfilzomib, lenalidomide, dexamethasone (Elo-KRd) in patients with no more than one prior line of therapy. Among the different combination therapies that STOMP study (NCT02343042) is evaluating, arm 9 includes patients receiving selinexor, dexamethasone, pomalidomide and elotuzumab (SPEd).

In conclusion, taking into consideration that in the near future a lot of MM patients will be treated with upfront daratumumab-based regimens, elotuzumab would be considered for RRMM setting, having a different mechanism of action. However, studies are needed to confirm the efficacy and safety of elotuzumab in these peculiar group of patients. 

## 3. Daratumumab

### 3.1. Daratumumab Monotherapy 

Daratumumab, the first fully human anti-CD-38 mAb evaluated for the treatment of MM, showed single-agent antitumor activity in the phase 1/2 GEN501 [34] and in the phase II SIRIUS [35] studies. After different doses of daratumumab were explored in the part 1 of GEN501 study [34] without identification of a maximum tolerated dose, the cohort treated with daratumumab at dose of 16 mg/kg in the part 2 of the study achieved an ORR of 36%. In the SIRIUS study [35], 106 patients with RRMM and a median of 5 previous lines of therapy (range 2–14) received daratumumab 16 mg/kg per week for 8 weeks, then every 2 weeks for 16 weeks and every 4 weeks thereafter. The ORR was 29% and after a median follow-up of 9.3 months, median PFS was 3.7 months. A pooled updated analysis of these studies [36] confirmed the significant activity of monotherapy with daratumumab in 148 heavily pretreated and highly refractory MM patients. The ORR for the combined data set was 31% (at least VGPR = 14%) and, after a longer follow-up of 20.7 months, median PFS and OS were 4 months, and 20 months, respectively. Notably, the median PFS was better in patients obtaining at least a PR compared to those with a lower response rate (15 months versus 3 months). Infusion-related reactions (IRRs), mainly consisting of nasal congestion, cough, allergic rhinitis, throat irritation and dyspnea, were documented in 48% of patients but they were of grade ≥3 only in 2.7% of them. Moreover, 96% of these events occurred during the first infusion whereas 7% developed during the second one. Final safety and efficacy results of the combined analysis of GEN501 and SIRIUS [37] have been recently published and show, after a median follow-up of 36.6 months a median OS of 20.5 months with a 3-year OS of 36.5%. The most frequent grade 3–4 side effects reported during treatment were anemia (18%), thrombocytopenia (14%), neutropenia (10%) and infections (9%). Safety profile of daratumumab monotherapy was evaluated in two multicenter early access treatment protocols (EAP) conducted in patients with ≥3 prior lines of therapy. In the first US study by Chari et al. [38], 348 RRMM patients received a median of 8 daratumumab infusions achieving an ORR of 23%. Grade 3–4 IRRs occurred in 8% of patients, mainly during the first infusion. In the Spanish study by Alegre et al. [39], 73 patients after a median of 12 daratumumab infusions achieved an ORR of 24.7% with PFS of 4 months. Only 2.7% of patients developed a grade 3–4 IRR. In a real-world setting [40]. However, daratumumab monotherapy showed little efficacy as reported by a retrospective analysis of 41 patients with a median age of 68 years who had received a median of 4 prior therapies. Despite an ORR of 23% similar to those reported in the GEN501 and SIRIUS studies, median PFS and OS were 1.9, and 6.5 months, respectively.

### 3.2. Daratumumab Plus IMiDs

Rationale for the combining daratumumab with lenalidomide was based on the in vitro synergistic activity between this mAb and IMiDs, starting from lenalidomide [41]. The phase I/II GEN503 study [42] assessed safety and activity of this triplet (DRd) in 32 patients with a median of 2 prior therapies (range 1–3) who received daratumumab 16 mg/kg (with the scheduleof SIRIUS study) plus lenalidomide (25 mg days 1–21) and dexamethasone (40 mg per week). The final results of this study have been recently published [43], and after a median follow-up of 32.5 months, ORR rate was 81% with 69% of patients achieving at least a VGPR and 44% a CR or better. Median PFS and OS were not reached and 2-year PFS and OS rates were 69%, and 78%, respectively. In relation to toxicities, most common ≥ grade 3 adverse events were neutropenia (84%) and thrombocytopenia (15.6%). 

The phase III POLLUX trial [39], with primary endpoint PFS, compared DRd versus Rd alone in 569 patients with RRMM who had previously received ≥1 prior line of therapy. Patients were randomized to Rd (lenalidomide 25 mg days 1–21 of each cycle plus dexamethasone 40 mg weekly) or DRd (Rd plus daratumumab 16 mg/kg every week in cycles 1, 2; every two weeks in cycles 3–6; every 4 weeks thereafter) and 28 days cycles were continued until progression or unacceptable toxicity. Approximately 85% and 18% of patients had been prior exposed to PI and lenalidomide, respectively, but patients refractory to lenalidomide were excluded. Patients who were enrolled in this study were not heavily pre-treated since they had received a median of one prior line of therapy. Updated efficacy data [44], after a median follow-up of 54.8 months, showed a significantly longer PFS for DRd group versus Rd group with a 56% reduction in the risk of progression or death (median 45 months versus 17.5 months; HR = 0.44; *p* < 0.0001). In patients with one prior line of therapy the PFS benefit was even greater with median PFS resulted to be 53.3 in DRd versus 19.6 months in Rd arm (HR = 0.42; *p* < 0.0001). Moreover remarkable efficacy was reported in bortezomib refractory patients treated with DRd (median PFS 34.3 months versus 11.3 months; HR = 0.42; *p* = 0.0008) and in those with high-risk cytogenetics (median PFS 26.8 months versus 8.3 months; HR = 0.37; *p* = 0.0056). Regarding response, significantly higher ORR was seen with DRd versus Rd (93% versus 76%) including ≥ VGPR (81% versus 49%) and ≥ CR (58% versus 24%; all *p* < 0.0001). A sustained Minimal Residual Disease (MRD) negativity at level of 10^−5^ ≥ 6 months and ≥12 months were documented in 20% and 16 patients receiving Drd, respectively (versus 2% and 1% in Rd group). Toxicity profile was similar across the two groups and major adverse events occurred in patients receiving DRd are reported in Table 1. Importantly, considering the median age of MM patients, we found that the results obtained in patients ≥75 years old were consistent with those reported in the overall population, and similar with the rate of grade 3–4 adverse events [45]. 

The ongoing phase III CONFIRM trial (NCT03836014), whose primary endpoint is OS at 4 years after randomization, is evaluating DRd administered continuously until progression disease versus a fixed duration of 24 months. 

The combination daratumumab, pomalidomide and dexamethasone (DPd) has been evaluated in the phase 1b EQUULEUS (MMY1001) study [46], assessing daratumumab in different regimens. One hundred and three patients with a median of 4 (range 1–13) prior therapies, 71% of whom refractory to PIs and IMiDs and 25% at high-risk cytogenetics received daratumumab (at the same dose and schedule of POLLUX trial), pomalidomide (4 mg on days 1–21) and dexamethasone (40 mg weekly). ORR was 60% with 42% of patients obtaining a VGPR or better and 17% a response ≥ CR. The responses were similar across patient subgroups including those with more than 3 lines of prior therapies and those refractory to PIs and IMiDs. After a median follow-up of 13 months, median PFS and OS were was 8.8 and 17.5 months, respectively. In the update of EQUULEUS study [47] after a follow-up of 24.7 months, ORR was 66%, median PFS 9.9 months and median OS 25 months, encouraging results considering the heavily pretreated study population. The most common grade 3–4 side effects, reported with this triplet, were neutropenia (77%), thrombocytopenia (19%), pneumonia (10%) [46]. DPd led to even better results in a less pretreated population enrolled in the arm B of the phase II MM-014 [48], including 112 patients with a median of one line of prior therapies (62.5% at first relapse). All patients have been treated with lenalidomide in the immediate prior line of therapy and 75% of them were lenalidomide refractory. Seventy eight percent of patients achieved ORR, 51% at least VGPR and 24% CR. After a median follow-up of 17.2 months, median PFS was not reached being 75% at 1 years. Notably, in patients lenalidomide, the refractory median PFS was 21.8 months and 1-year PFS 72%. These results showed the benefit of continuing immunomodulation with pomalidomide immediately after lenalidomide, even in case of failure of lenalidomide. Recently, Pierceall et al. [49], analyzing immunophenotipic changes in peripheral blood of patients receiving DPd from MM-14 study, demonstrated enhanced activation/differentiation of B, T and NK cells that is exhibited also in lenalidomide refractory patients. These data could explain the efficacy of DPd in patients heavily pre-treated who are refractory to both, daratumumab and pomalidomide as individual lines of therapy, reported by Emory group [50]. The ongoing phase III APOLLO trial (NCT03180736) comparing DPd versus Pd will address the effects of the addition of daratumumab to Pd in patients with RRMM who have received at least one treatment regimen.

### 3.3. Daratumumab Plus PIs

Daratumumab was assessed in combination with bortezomib and dexamethasone (DVd) in the phase III CASTOR trial [28] in which Vd (bortezomib 1.3 mg/m^2^ on days 1, 4, 8, 11; dexamethasone 20 mg on days 1, 2, 4, 5, 8, 9, 11, 12) given for 8 cycles was compared against DVd (daratumumab 16 mg/kg days 1, 8 and 15 during cycles 1 to 3, once every 3 weeks during cycles 4 to 8 and once every 4 weeks thereafter until progression). The study included 498 patients with RRMM who had previously received a median of two therapies and approximately one-half of patients had been exposed to PIs and IMIDs. IN relation to renal function, the enrolment of patients with a creatinine clearance >20 mL/min was allowed. At the last update [51], after a median follow-up of 50.2 months, median PFS was significantly longer in DVd group versus Vd (16.7 months versus 7.1 months; HR = 0.31; *p* < 0.0001) and this benefit was particularly relevant in patients treated with DVd at first relapse since median PFS resulted 27 months versus 7.9 months (HR = 0.21; *p* < 0.0001). In patients refractory to lenalidomide (any prior line) median PFS was 7.8 versus 4.9 months (HR = 0.44; *p* = 0.0002).

The ORR also improved (85% versus 63%), as did the rate of VGPR or better (63% versus 29%) and CR or better (30% versus 10%) (all *p* < 0.0001). Moreover, patients achieving a sustained MRD-negativity at level of 10^−5^ was significantly higher in the DVd arm since in 10% of patents it lasted at least 6 months (versus 1% in Vd group) and in 7% lasted at least 12 months (versus 0 in Vd group). The most common grade 3 or 4 adverse events are summarized in Table 1. As seen in POLLUX trial most IRRs occurred during the first infusion and were grade 1 or 2.

Phase 1b EQUULEUS study (MMY1001) [52], besides DPd, evaluated the combination daratumumab (16 mg/kg weekly during cycles 1, 2, every two weeks cycles 3–6 and every 4 weeks thereafter), carfilzomib (20 mg/m^2^ initial dose escalated to 70 mg/sm weekly) and dexamethasone (40 mg weekly) (DKd) in 85 patients with RRMM. Median number of prior therapies was 2 (range 1–4) and 60% of patients were refractory to lenalidomide. After a median follow-up of 16.6 months, ORR was 84% with 71% of patients achieving at least VGPR. The median PFS was not reached in the all-treated population, but it was 25.7 months in patients refractory to lenalidomide. As regard safety profile, the most frequent grade 3–4 hematologic adverse events were thrombocytopenia (31%) and neutropenia (21%). Grade 3–4 infections occurred in 19% of patients and consisted in pneumonia in 5% of cases. Of note, 10 patients (12%) developed grade 3–4 cardiac events, mainly cardiac failure, that resolved in 8 of them. The efficacy of this triplet has been confirmed in the phase III CANDOR [29,53] in which 466 RRMM patients were randomized to receive Kd (carfilzomib on days 1, 2, 8, 9, 15, 16 at dose of 20 mg/m^2^ on days 1 and 2 of cycle 1 and 56 mg/m^2^ thereafter; dexamethasone 40 mg weekly) or DKd (Kd plus daratumumab 16 mg/kg with the same schedule of MMY1001 study). Patients had received a median of 2 prior therapies (range 1–2) and 33% were lenalidomide refractory. After a median follow-up of 16.9 months, median PFS, primary endpoint of the study, was not reached in the DKd group and 15.8 in the Kd group (HR = 0.63; *p* = 0.0027). The benefit was observed in all subgroups of patients including those refractory to lenalidomide (HR = 0.47), whereas PFS HR was lower in the bortezomib-refractory group (HR = 0.84). The response rate was significantly higher in patients treated with DKd in terms of ORR (84% versus 75%; *p* = 0.0080) and at least VGPR (69% versus 49%). Moreover 18% of patients in DKd group achieved a MRD rate at 12 months of 18% versus 4% in the Kd group. The most common grade 3–4 toxicities are pictured in Table 1.

In the ongoing phase II DARIA study [54], conducted by the Greek Myeloma Study Group, daratumumab was evaluated, in combination with ixazomib and dexamethasone (IDd), in patients who have received one prior treatment with a lenalidomide-based regimen. Very preliminary results presented at the last EHA Congress showed promising response rates. Another phase II multicenter study is testing IDd (NCT03439293), whereas another one by MD Anderson Cancer Center is evaluating IDd after 3 cycles of DVd in RRMM (NCT03763162).

Phase II studies with quadruplets containing daratumumab plus pomalidomide, carfilzomib and dexamethasone are ongoing (NCT01665794, NCT04176718), whereas preliminary safety data of daratumumab combined with pomalidomide, ixazomib and dexamethasone showed good tolerability and activity [55].

### 3.4. Daratumumab Plus Venetoclax 

An interesting combination under evaluation including daratumumab is that with venetoclax, a selective and potent oral BCL-2 inhibitor that induces apoptosis in MM cell lines and primary samples, particularly those with t(11;14), a cytogenetic abnormality documented in near 20% of MM patients. Moreover, clinical studies demonstrated efficacy of venetoclax in combination with bortezomib and dexamethasone in RRMM [56]. An ongoing phase I/II study [57] is assessing safety and efficacy of venetoclax, daratumumab, dexamethasone with or without bortezomib in RRMM. Twenty-four Patients with t(11;14) and at least one prior line of therapy were treated with venetoclax, daratumumab and dexamethasone (VenDd) in part 1 of study whereas part 2 included 24 patients irrespective of cytogenetics, with 1–3 prior lines of therapy, who received venetoclax, daratumumab, bortezomib and dexamethasone (VenDVd). ORR was 96% with triplet and 92% with quadruplet combination, being ≥ CR rates 54% and 42%, respectively. Remarkably, 21% of patients with t(11;14) who received VenDd obtained MRD negativity at level of 10^−5^. The most important grade 3–4 adverse events were infections occurring in 21% of patients in the VenDd group and 17% in the VenDVd one. At 12 months, no patients treated with venetoclax at dose of 800 mg daily had progressive disease.

### 3.5. Daratumumab Plus Selinexor

Promising results in RRMM (≥3 prior lines of therapy) have been obtained, combining daratumumab with selinexor, the first-in-class oral Selective Inhibitor of Nuclear Export (SINE) to be approved with dexamethasone for advanced disease [58]. In a phase Ib/2 study [59] selinexor, in combination with daratumumab and dexamethasone (SDd), was tested at dose of 100 mg weekly or 60 mg twice-weekly; maximum tolerated dose and recommended phase II dose of SDd was found to be selinexor 100 mg, daratumumab 16 mg/kg and dexamethasone 40 mg, administered weekly. Overall, 34 patients with a median of 3 prior therapies were enrolled. Most common grade 3–4 adverse events were thorombocytopenia (32%), neutropenia (26%), fatigue (18%) and nausea (95). ORR was 73% and median PFS 12.5 months. An ongoing phase II study by PETHEMA (SELIBORDARA, NCT03589222) is assessing the quadruplet selinexor, bortezomib, daratumumab and dexamethasone in patients who have received at least 3 prior lines of therapy. 

### 3.6. Intravenous Versus Subcutaneous Daratumumab 

Despite the good safety profile, daratumumab, is administered as an intravenous formulation (IV) needing a long infusion time, being 7.0 h for the first infusion, 4.3 h for the second infusion and 3.5 h for subsequent administrations. However, a shorter duration of infusion could result in a reduction of nursing time for each patient, as well as in optimizing the requested time for patients care. For these reasons, daratumumab has been tested as subcutaneous formulation (SC) in 3 clinical trials. The first one was phase 1b dose-escalation PAVO study [60], evaluating safety and PK profile of daratumumab administered sc in combination with the recombinant human hyaluronidase PH20 enzyme (rHuPH20) at dose of 1200 (8 patients) or 1800 mg (45 patients). IRRs occurred mainly during the first infusion in 12.5% and 24.4% of patients receiving 1200 mg, and 1800 mg, respectively, and were generally of grade 1–2. In relation to grade 3–4 adverse events, neutropenia, thrombocytopenia, upper respiratory infections and pneumonia developed in 12.5% each in 1200 mg group patients versus 6.7, 6.7%, 0, and 4.4%, respectively, in 1800 mg group. The 1800 mg dose was comparable in term of PK profile with daratumumab 16 mg/kg IV dose. ORR rates were 42.2%, including 8.9% sCR, in patients receiving 1800 mg versus 25% in those 1200 mg. Parte 2 of PAVO study [61] evaluated a concentrated, pre-mixed co-formulation of daratumumab 1800 mg plus rHuPH20 (DARA SC) administered to 25 patients with a median of 3 (range 2–9) prior lines of therapy. DARA SC was given weekly during cycles 1 and 2, every two weeks during cycles 3–6 and every 4 weeks thereafter. Daratumumab serum concentration following DARA SC was consistent with IV daratumumab as well similar was safety profile. After a median follow-up of 14.2 months, ORR with DARA SC was 52% with 28% of patients achieving VGPG. Moreover median PFS was 12 months for all patients and 11.7 months for those refractory to both PIs and IMiDs. Based on these results, the phase III COLUMBA trial [62] tested the non-inferiority for ORR of daratumumab sc versus daratumumab iv. A total of 522 patients with ≥3 prior lines of therapy were randomized to receive daratumumab sc 1800 mg plus rHuPH20 2000 U/mL or conventional daratumumab iv with the same schedule of PAVO study. After a median follow-up of 7.5 months, primary end-point was met since ORR was 41% in the sc group versus 37% in the iv group. The rates of at least VGPR (19% versus 17%, respectively) and median PFS (5.6 versus 6.1 months, respectively) were similar.

This trial demonstrated that a subcutaneous formulation of daratumumb, needing five minutes for delivery, maintains the same efficacy and safety of original formulation. The ongoing phase II PLEIADES study [63] is assessing daratumumab sc, in combination with standard care in 3 cohorts of both newly diagnosed and RRMM patients. In RRMM cohort, 65 patients received daratumumab sc wth Rd (D-Rd) obtaining an ORR of 90.8% and at least VGPR of 64.6% with less than 5% of patients with toxicities requiring treatment discontinuation. An ongoing randomized phase II study (NCT03871829; LYNX) will evaluate the efficacy and safety of retreatment with daratumumab sc in patients with RRMM previously exposed to daratumumab iv.

In Table 2 we summarized the ongoing clinical studies with daratumumab in RRMM.

## 4. Isatuximab

### 4.1. Isatuximab Monotherapy 

Isatuximab, an IgG1k chimeric monoclonal antibody directed to CD38, appears to be a strong blocker of the multiple enzymatic functions of the target molecule [64]. It binds to a specific epitope on the human cell surface antigen CD38, which is widely and uniformely expressed on myeloma cells, and it leads to apoptosis of MM cells without crosslinking of the Fc receptors of the antibody [65]. Several clinical trials demonstrated the efficacy of isatuximab in RRMM both in monotherapy and in association with other drugs. 

A phase I multicenter dose-escalation study [66] evaluated safety and toxicity of isatuximab monotherapy given at dose ranging from 0.0001 mg/kg to 20 mg/kg in RRMM patients. Overall, 84 patients with a median of 5 (range 1–13) prior lines of therapy were enrolled and 62% of them had received prior carfilzomib or pomalidomide. Maximum tolerated dose (MTD) was not reached and IRRs developed in 47.6% of patients during the first cycle, being of grade 1 and 2 in 94% of cases. As regard efficacy, in patients treated with isatuximab at dose 10–20 mg/kg ORR was 24% and median PFS 3.7 months, consistent with that reported with daratumumab monotherapy. These results have been confirmed in a phase II multicenter randomized study [67] in which patients who had received three or more prior lines of therapy were allocated to receive 4 different doses and schedules of isatuximab as follows: 3 mg/kg every 2 weeks, 10 mg/kg every two weeks, 10 mg/kg every two weeks for 2 cycles and every 4 weeks thereafter, 20 mg/kg weekly during the first cycle and every two weeks thereafter. Overall, 97 patients with a median of 5 (range 2–14) prior lines of therapy were enrolled and among them 83% and 64% were refractory to lenalidomide and pomalidomide, respectively, as well as 74% and 61% were refractory to bortezomib and carfilzomib, respectively. At dose ≥10 mg/kg ORR was 24.3% with 15% of patients achieving a VGPR, median PFS was 4.6 months and median OS was 18.7 months. The part 2 of the same study has been recently published [68]. Patients treated with ≥3 prior lines were randomized to receive isatuximab 20 mg/kg weekly for 4 infusions followed by 20 mg/kg every 2 weeks either as monotherapy (Isa: 109 patients) or in combination with dexamethasone (Isa-dex: 55 patients) 40 mg weekly. The median number of prior lines of therapy was 4 (range 2–10) in both arms. ORR was 23.9% and 43.6% in Isa and Isa-dex arm, respectively (*p* = 0.008). As regard outcome measures, median PFS and OS were 4.9 and 18.9 months for Isa group and 10.2 and 17.3 for Isa-dex group. IRRs occurred in 40% of both groups of patients, mainly of grade 1–2, whereas grade 3–4 neutropenia and infections were the most common toxicities.

### 4.2. Isatuximab Plus IMiDs

As well as for elotuzumab and daratumumab, also isatuximab was tested in combination with lenalidomide and dexamethasone in a phase 1b dose escalation study [69]. Patients were treated with different doses and schedules of isatuximab with the aim of determining the maximum tolerated dose of isatuximab combined with lenalidomide (25 mg days 1–21) and dexamethasone (40 mg weekly) (Isa-Rd). A total of 57 patients were enrolled; they had received a median of 5 (range 1–12) previous lines therapies, 88% were refractory to any IMiDs-based therapies, 65% to bortezomib and 92% to carfilzomib. The MTD was no reached and the selected dose of isatuximab for further studies evaluating this triplet was 10 mg/kg weekly during cycle 1 and then every two weeks. After a median follow-up of 9 months, ORR for the all population was 51% with a median PFS of 8.5 months. ORR was 52% in lenalidomide-refractory patients and 48% in those who had received ≥3 previous treatment lines. IRRs occurred in 56% of patients, but they were grade 1–2 in 84%. Moreover, the most common grade 3–4 adverse events were neutropenia (60%), thrombocytopenia (38%) and pneumonia (9%). Mikhael et al. [30] found an ORR of 62%, with median PFS of 17.6 months, in a phase Ib dose-escalation study of isatuximab, in association with pomalidomide (4 mg days 1–21) and dexamethasone (40 mg weekly). Also in this study MTD was not reached. Among 45 patients enrolled with a median of 5 prior lines of therapy (range 3–12), 82% were lenalidomide-refractory and the ORR was 56.8%. The incidence of IRRs was similar to that reported with Isa-Rd but the incidence of grade ≥3 neutropenia was higher (84%), as well as that of pneumonia (18%). Based on the promising data from this combination in a very heavily pretreated RRMM population, phase III ICARIA trial [70] compared Isa-Pd versus Pd in 307 RRMM who had previously received ≥2 lines of therapy (median 3, range 2–4). Overall, all patients were previously treated with lenalidomide and proteasome inhibitors, being 93% refractory to lenalidomide and 76% to at least one PI. Treatment consisted of pomalidomide 4 mg days 1–21 and dexametahsone 40 mg weekly in the Pd group with the addition of isatuximab 10 mg/kg weekly in the first cycle and every two weeks thereafter in the Isa-Pd one. After a median follow up of 11.6 months, a 41% reduction of the risk of disease progression or death was reported in Isa-Pd versus Pd group, with a median PFS of 11.5 versus 6.5 months, respectively (HR = 0.59; *p* = 0.001). This benefit was conserved in all subgroups, in particular in lenalidomide-refractory patients (HR = 0.59), high-risk cytogenetic MM (HR = 0.66) and patients with impairment of renal function (HR = 0.50). ORR and ≥ VGPR were 63% and 32% versus 32% and 9% in Isa-Pd and Pd group, respectively. As for safety profile, IRRs were the most relevant adverse events occurring in 38% of patients in Isa-Pd group, 2% of which was grade 3–4. Other grade 3–4 toxicities are reported in Table 1.

The benefits in terms of PFS and ORR was observed in patients ≥75 years old, as shown in a pre-specified subgroup analysis of ICARIA trial [71] comparing Isa-Pd versus Pd, in three age groups as follows: <65 years old, 65–74 and ≥75 years old. The median PFS was significantly longer with Isa-Pd and similar between three groups (11.5 versus 11.5, versus 11.4, respectively). However, older patients showed a higher rates of serious treatment-emergent adverse events with discontinuation of therapy either in Isa-Pd and in Pd arm. 

Another subgroup analysis of ICARIA trial [72] analysed outcome of patients with renal impairment (RI) defined as estimated glomerular filtration rate (eGFR) <60 mL/min/1.73 m^2^). Median PFS was 9.5 versus 3.7 months in patients with RI receiving Isa-Pd, and Pd, respectively (HR= 0.50) whereas in patients without RI median PFS was 12.7 versus 7.9 months, respectively (HR = 0.58). Moreover, compared with Pd the addition of isatuximab improved the complete renal response (71.9% versus 38.1%) with a median time to renal response of 3.4 weeks in Isa-Pd versus 7.3 weeks in Pd group. 

There were no differences in the IRRs rate between patients with, and without, RI, and the most frequent grade 3–4 non-hematologic adverse events in patients receiving Isa-Pd were infections and pneumonia. In conclusion, Isa-Pd represents a valuable treatment option for patients with RRMM presenting with renal dysfunction, considering that it is not necessary a dose adjustment, differently from lenalidomide. 

Finally, benefit of Isa-Pd over Pd was documented also in patients with isolated gain (1q21) as showed in a retrospective analysis from patients enrolled in ICARIA and phase Ib study [73].

### 4.3. Isatuximab Plus PIs

After a phase 1b study [31] established the feasibility and safety of isatuximab, combined with carfilzomib, the phase III IKEMA trial [74] compared isatuximab, carfilzomib, dexamethasone (Isa-Kd) with carfilzomib, dexamethasone (Kd) in RRMM patients who had received 1–3 previous lines of therapy. At last EHA Congress, Moreau presented results of an interim analysis. Three hundred two patients were randomized to receive Kd (carfilzomib 20 mg/m^2^ days 1 and 2 of cycle 1, 56 mg/m^2^ days 8, 9, 15, 16 of cycle 1 and subsequent cycles plus dexamethasone 20 mg days 1, 2, 8, 9, 15 and 16) or Kd plus isatuximab (10 mg/kg weekly during cycle 1 and every two weeks thereafter). Patients had received a median of 2 prior lines of therapy (range 1–4), 45% were refractory to IMidDs and 33% to bortezomib. After a median follow-up of 20.7 months median PFS not reached in the Isa-Kd group versus 19.15 months in the Kd one (HR = 0.53; *p*< 0.0007). This benefit was confirmed among all the subgroups of analysis, in particular HR was 0.59 in lenalidomide-refractory patients, 0.56 in patients previously receiving bortezomib and 0.72 in high-risk cytogenetic subgroup. As for data about OS it is necessary a longer follow-up. No significant difference was found between two arms as regard ORR (86.6% versus 82.9 in Isa-Kd and Kd group, respectively) whereas high quality responses were more frequent in Isa-Kd patients (VGPR or better 72.6% versus 56%). In these latter MRD negativity by NGS at level of 10^−5^ was documented in 41% of patients treated with IsaKd versus 23% with Kd. 

IRRs occurred mostly during the first infusion and were grade ≥3 in less than 1% of patients. Grade 3–4 adverse events are summarized in Table 1.

As well as for daratumumab, an ongoing phase II study (NCT04287855) is evaluating isatuximab plus pomalidomide, carfilzomib and dexamethasone. Moreover, a phase Ib study (NCT04045795) is assessing safety and tolerability of isatuximab administered subcutaneously versus intravenously.

**Figure 1:**
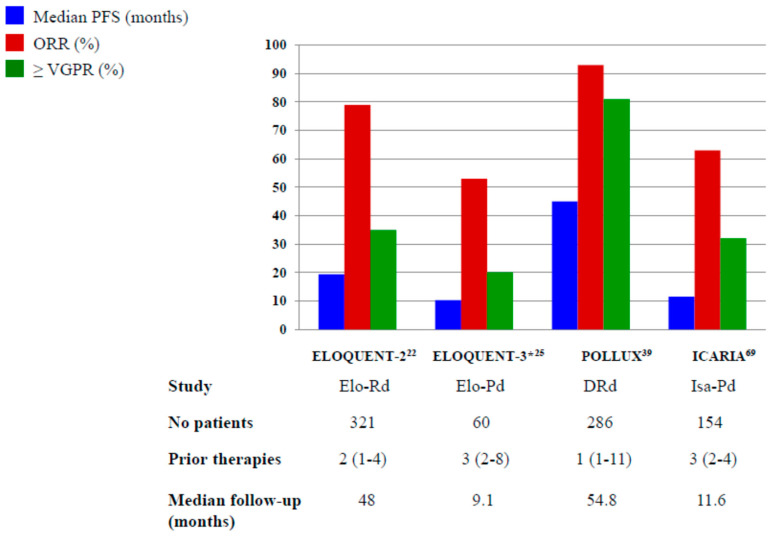
Results of Phase III and randomized phase II* trials with elotuzumab, daratumumab and isatuximab plus IMiDs. Elo-Rd: elotuzumab, lenalidomide, dexamethasone; Elo-Pd: elotuzumab, pomalidomide, dexamethasone; DRd: daratumumab, lenalidomide, dexamethasone; Isa-Pd: isatuximab, pomalidomide, dexamethasone.

**Figure 2:**
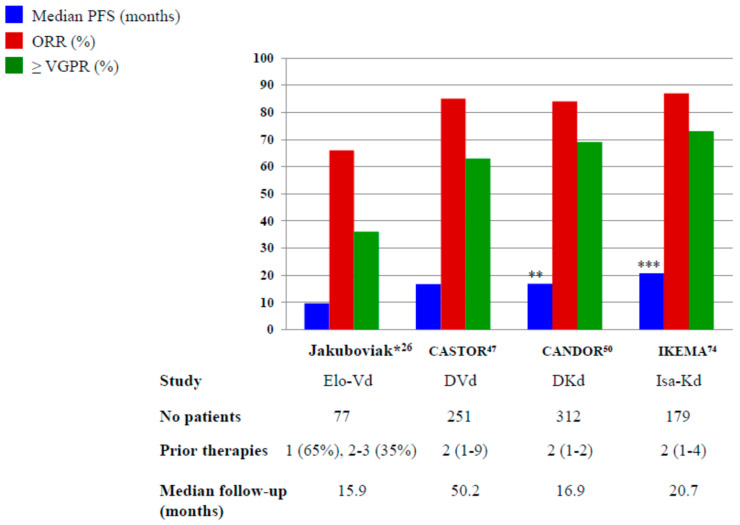
The results of Phase III and randomized phase II* trials with elotuzumab, daratumumab and isatuximab plus PIs. Elo-Vd: elotuzumab, bortezomib, dexamethasone; DVd: daratumumab, bortezomib, dexamethasone; DKd: daratumumab, carfilzomib, dexamethasone; Isa-Kd: isatuximab, carfilzomib, dexamethasone ** Not reached after a median follow-up of 16.9 months *** Not reached after a median follow-up of 20.7 months.

In Figure 1 and Figure 2, we pictured the results of main phase III clinical trials including elotuzumab, daratumumab and isatuximab.

## 5. Conclusions and Perspectives

Immunotherapies like as MoAbs are becoming the major players in the treatment of MM patients. In the RRMM patients triplets, including elotuzumab and daratumumab were found to be superior to doublet standard regimens and they are bound to rapidly change the outcome of RRMM patients. Although, the overall survival data are still immature for mostly phase III studies, in the ELOQUENT-2 trial elotuzumab, in combination with lenalidomide and dexamethasone, demonstrated significant improvement in overall survival after a median follow-up of almost six years. A third monoclonal antibody, isatuximab, was recently approved for RRMM setting by FDA. The benefit given by the triplet containing MoAbs, compared with doublet drug combinations, is also consistent in the subset of patients with high-risk cytogenetics, advanced ISS stage and in older patients. Monoclonal antibodies have shown a good safety profile and recent approval of subcutaneous daratumumab will improve quality of life for many patients. However, the increasing use of MoAbs upfront will probably make the treatment of RRMM more problematic but novel immunotherapeutic approaches as CAR-T cells, bispecific antibodies (BiTEs) and antibody-drug conjugates are coming into play and the outcome of MM patients is expected to continue to improve.

## Figures and Tables

**Table 1:** Grade 3–4 adverse events (%) reported in randomized phase II and phase III trials with elotuzumab, daratumumab and isatuximab.

Treatment	Neutropenia	Thrombocytopenia	Infections	Pneumonia	Cardiac Disorders	Vascular Events	Neuropathy
ELOQUENT-2 [22] Elo-Rd versus Rd	36 versus 45	21 versus 21	33 versus 26	14 versus 10	5 versus 8	10 versus 8	0
ELOQUENT-3 [25] Elo-Pd versus Pd	13 versus 27	8 versus 5	13 versus 22	5 versus 9	7 versus 4	3 versus 0	0
Jakubowiak [26] Elo-VD versus VD	0	9 versus 17	21 versus 13	8 versus 4	0 versus 1	0	9 versus 12
POLLUX [27] DRd versus Rd	52 versus 37	12.7 versus 13.5	28.3 versus 22.8	7.8 versus 8.2	0	0	0
CASTOR [28] DVd versus Vd	12.8 versus 4.2	45.3 versus 32.9	21.4 versus 19	8.2 versus 9.7	0	6.6 versus 0.8 *	4.5 versus 6.8
CANDOR [29] DKd versus Kd	9 versus 6	24 versus 16	29 versus 16	13 versus 9	7 versus 12	18 versus 13 *	1 versus 1
ICARIA [30] Isa-Pd versus Pd	85 versus 70	31 versus 25	6 versus 1	16 versus 14	0	0	0
IKEMA [31] Isa-Kd versus Kd	19.2 versus 23.8	30 versus 23.8	6 versus 2	16.4 versus 12.3	4 versus 4	20.3 versus 19.7 *	0

Elo-Rd: elotuzumab, lenalidomide, dexamethasone; Elo-Pd: elotuzumab, pomalidomide, dexamethasone; Elo-VD: elotuzumab, bortezomib, dexamethasone; DRd: daratumumab, lenalidomide, dexamethasone; DVd: daratumumab, bortezomib, dexamethasone; DKd: daratumumab, carfilzomib, dexamethasone; Isa-Pd: isatuximab, pomalidomide, dexamethasone; Isa-Kd: isatuximab, carfilzomib, dexamethasone. *: Hypertension.

**Table 2:** Ongoing clinical trial with daratumumab in RRMM.

Study	Phase	Treatment	NCT Identifier
Study of ciforadenant in combination with daratumumab in patients with relapsed or refractory multiple myeloma	I	Ciforadenant 100 mg orally twice daily plus daratumumab 16 mg/kg mg iv weekly cycles 1 and 2, every two weeks cycles 3–6 and every 4 weeks thereafter	04280328
Study of melphalan flufenamide (Melflufen) + dex with bortezomib or daratumumab in patients with RRMM (ANCHOR)	I/II	Melflufen 30 mg and 40 mg or 20 mg in day 1 plus daratumumab 16 mg/kg weekly for 8 doses, every other weeks for 8 doses and then every 4 weeks plus dexamethasone or melfuflen (same schedule) plus bortezomib 1.3 mg/sm sc days 1, 4, 8, 11 and dexamethasone	03481556
INCB001158 combined with subcutaneous (SC) daratumumab, compared to daratumumab sc, in relapsed or refractory multiple myeloma	I/II	INCB001158 orally twice daily with dose escalation, plus daratumumab sc 1800 mg weekly cycles 1 and 2, every two weeks cycles 3–6 and every 4 weeks thereafter versus daratumumab sc	03837509
Daratumumab, azacitidine, and dexamethasone for treatment of patients with recurrent or refractory multiple myeloma previously treated with daratumumab	II	Azacitidine iv for 5 days plus daratumumab 16 mg/kg iv weekly for 2 cycles, every 2 weeks for 4 cycles and every 4 weeks thereafter plus dexamethasone	04407442
A study to determine the efficacy of the combination of daratumumab plus durvalumab (D2) in subjects with relapsed and refractory multiple myeloma (FUSION-MM-005)	II	Durvalumab iv 1500 mg on day 2 cycle 1 and on day 1 thereafter plus daratumunìmab 16 mg/kg iv weekly cycles 1 and 2, every two weeks cycles 3–6 and every 4 weeks thereafter versus daratumumab sc	03000452
A study of JNJ-63723283, an anti-programmed death-1 monoclonal antibody administered in combination with daratumumab, compared with daratumumab alone in partecipants with relapsed or refractory multiple myeloma	II/III	Daratumunìmab 16 mg/kg iv weekly cycles 1 and 2, every two weeks cycles 3–6 and every 4 weeks thereafter plus JNJ-63723283 240 mg iv week 1 on cycle 1 day 2, cycle 1 day 15 then every 2 weeks thereafter versus daratumumab iv	03357952
Evaluation of efficacy and safety of belantamab mafodotin, bortezomib and dexamethasone versus daratumumab, bortezomib and dexamethasone in partecipants with relapsed/refractory multiple myeloma (DREAMM 7)	III	Belantamab mafodotin plus bortezomib and dexamethasone versus daratumumab, bortezomib and dexamethasone (DVd)	04246047
A study comparing JNJ-68284528, a CAR-T therapy directed against B-cell Maturationa Antigen (BCMA), versus pomalidomide, bortezomib and dexametahsone (PVd) or daratumumab, pomalidomide and dexamethasone (DPd) in partecipants with relapsed and lenalidomide-refractory multiple myeloma (CARTIDUDE-4)	III	Pomalidomide 4 mg days 1–14 plus bortezomib 1.3 mg/m^2^ days 1, 4, 8, 11 (cycles 1–8) and days 1 and 8 thereafter plus dexamethasone (PVd) or daratumumab 1800 mg sc weekly cycles 1 and 2, every two weeks cycles 3–6 and every 4 weeks thereafter plus pomalidomide 4 mg days 1–21 plus dexamethasone (DPd) versus JNJ-68284528 CAR-T therapy	04181827
Efficacy and safety study of bb2121 versus standard regimens in subjects with relapsed and refractory multiple myeloma (RRMM) (KarMMa-3)	III	Daratumumab, pomalidomide, dexamethasone (DPd) or daratumumab, bortezomib, dexamethasone (DVd) or ixazomib, lenaliodmide, dexametasome (IxaRd) or carfilzomib, dexamethasone (Kd) or elotuzumab, pomalidomide, dexamethasone (EPd) versus bb2121	03651128
